# Can palm oil be substituted? A rheological study of fat mixtures

**DOI:** 10.3389/fnut.2024.1388259

**Published:** 2024-10-03

**Authors:** Anikó Kovács, Szabolcs Homolya, Ágoston Temesi, Brigitta Unger-Plasek, Tímea Kaszab, Katalin Badak-Kerti

**Affiliations:** Hungarian University of Agriculture and Life Sciences, Gödöllő, Hungary

**Keywords:** palm oil, palm oil replacement, palm oil substitution, rheology, fat mixture

## Abstract

**Introduction:**

Due to its distinctive fatty acid makeup, which makes it simple to utilize in various applications, palm oil is one of the most widely used fats in the food business. However, the increased palm oil production is causing global environmental issues, including the loss of rainforests. As a result, companies that use palm oil, particularly the food industry, must hunt for alternate sources of fat that can replace palm oil. Oleogels, which are created from liquid oils, are one of the potential substitutes. Another alternative is to combine solid fat and liquid oil, however, research in this area is scarce.

**Methods:**

To fill this gap, we used a fully hydrogenized rapeseed oil and sunflower oil mixture in three different ratios for our research. We examined the rheological behavior of the mixtures using oscillatory and rotational viscometry.

**Results and discussion:**

According to our results, 35% FH rapeseed oil sample yielded results most closely resembling the control palm mid fraction. Our findings show that replacing palm oil with a combination of completely hydrogenated rapeseed oil and sunflower oil is only viable to a limited degree from a rheological point of view.

## Introduction

Fats and oils are important components of processed foods, they are wildly used, and, in most recipes, they make up a significant part of the food. However, the types of fats and oils used have changed over the years due to changes in consumer attitudes and lifestyles. A healthy lifestyle and the effects of processed meals on the environment are becoming more and more important to consumers (1). They are searching for food products with low levels of saturated fat and no trans-fat. The food manufacturers had to create new food formulas to meet the increasing needs. Palm fat was one of the essential components used to replace lipids containing trans fatty acids (2). The use of palm fat has increased because of its distinct fatty acid content, good oxidation stability, and the variety of fractions that may be extracted from it (3–5). In 1961, 1.5 million tonnes were produced, in 2011, it’s grown to 48.6 million tonnes (6). The world’s first five palm oil-producing countries are Indonesia, Malaysia, Thailand, Columbia, and Nigeria. From this, the first two give 85% of the word productions (4). The oil palm yield is limited to wet tropical areas; therefore, the increasing production is going at the expense of rainforests. In 2005, over 50% of oil palm plantations in Indonesia and Malaysia were established in regions that were tropical rainforests in 1990 (7). These palm oil plantations are reducing the Earth’s resilience against the increasing greenhouse effect and global warming. Also, it is reducing biodiversity, and many species are in danger, like orangutans. Because most of the countries produce palm fat for export the transportation of it also increases the negative environmental impact (8). These serious issues lead to the demand for palm oil-free products in the food market. However, if we examine raw materials that could substitute for palm oil, we find very limited options (2).

One solution for palm oil substitution is the use of oleogels. The term “oleogel” is a new definition for making liquid oils with gel-like structures. The benefits of these ingredients are that they have characteristics like solid fats (rheology, viscoelasticity, texture, and so on) without containing a huge amount of solid fat (9–12). The oleogels are complex microstructural systems in which the organic liquid is closed in a heat-reversible, three-dimensional gel structure with a gel-forming material. Different plant oils can be used for this purpose, for example, sunflower oil, rapeseed oil, and olive oil (13). The gel-forming material must come from a natural source, satisfy the food safety regulations, and be lipophilic, heat-reversible, and surfactant (14–16). These materials could be plant waxes, mono- and diglycerides, alcohols, esters of fatty acids, phospholipids, and phytosterols (17). According to research, Oleogels were successfully applied to bakery products (18), chocolate products (19), and meat products (20) to reduce the saturated fatty acid content without significant changes in the quality of the products. However, more research is needed to identify the application areas of these materials.

Other options for the palm oil substitution are mixing full hydrogenated oils with non-hydrogenated liquid oils. By mixing rapeseed, sunflower, or soy oil with fully hydrogenized oil, a good tailor-made confectionary fat can be achieved (21). The melting profile will be flatter, especially at lower temperatures. However, between 20 and 40°C, the melting profile can be very similar to that of palm fat. Also, these mixtures, due to their higher liquid fat content will have a 15% lower saturated fatty acid content than palm fat. If we want to produce a tailor-made confectionary fat, then the full hydrogenated fat should be mixed with lauric fat, like coconut fat. This combination results in a steeper melting profile and a higher solid fat content between 10 and 20°C. It will have a pleasant melting behavior at body temperature, which could result in further benefits. However, this mixture will have a higher saturated fatty acid content, which – according to *Hinrichsen* -could be a problem for consumers (2).

The third alternative could be the use of tropical fats like cocoa butter, shea, sal, illipe, kokum and mango seed fat. These fats have a relatively high symmetric triglyceride content, as a result, they must be tempered before being used to have a right stable crystal form. This makes their use more difficult and expensive. To avoid the tempering, the tropical fat must be esterified. Esterification makes some part of the symmetric triglycerides asymmetric, which eliminates the requirement for tempering in the production process. The trans esterified tropical fats have a high solid fat content in a relative huge range, their melting point is higher than the body temperature, hence for using it in food industry addition of other oil is required. In this case for the best melting profile also the lauric fats will be beneficial. This mixture will have a high solid fat content between 10 and 20°C, while at body temperature it is almost liquid. However, it will also have a higher saturated fatty acid content (2).

Sometimes, only liquid oils (sunflower, rapeseed, etc.) can be used in place of palm fat. This might be useful for products that employ a little bit of palm oil or the only liquid portion (olein fraction) of it. These oils have a lower saturated fatty acid content which is beneficial for the health, however from technological point of view it is negative, because these oils have lower oxidation stability (22).

Based on the research findings mentioned earlier, substituting palm oil is a highly complex issue that requires case-by-case analysis. Our research focused specifically on its application in confectionery, examining the substitution of the palm mid fraction, which is maybe the most used type of fat in such applications. To develop a potential palm oil substitute, we selected fully hydrogenated and non-hydrogenated liquid fats in various mixing ratios, guided by Hinrichsen’s (2) research. We tested three different mixing ratios of fully hydrogenated rapeseed oil and sunflower oil.

Both fats originate from continental sources, thereby mitigating some of the socio-economic constraints associated with the use of tropical fats. The goal of our study was to identify which mixing ratio most closely replicates the techno-functional attributes of palm oil. In confectionery applications, such as creams and fillings, one of the most critical techno-functional properties is viscosity (23). Consequently, we analyzed the rheological behavior of the mixtures and compared it to that of the control palm fat fraction.

## Materials and methods

In our research commercially available palm mid fraction (PMF) was used as a control sample. Based on the literature we chose fully hydrogenised rapeseed oil (as the hard fat part) and sunflower oil mixtures to investigate the opportunities of the PMF substitution. All samples were provided by Bunge Ltd.

We prepared three distinct fat mixtures with varying weight ratios of fully hydrogenated (FH) rapeseed oil and sunflower oil, as detailed below:

- 25% FH rapeseed +75% sunflower oil (R25NO75).
- 30% FH rapeseed +70% sunflower oil (R30NO70).
- 35% FH rapeseed +65% sunflower oil (R35NO65).

For sample preparation, the fully hydrogenated rapeseed oil was melted in an 80°C water bath until fully liquefied. Following this, sunflower oil at room temperature was added according to the specified mixing ratio, and the two fats were mixed at room temperature until a homogeneous mixture was achieved. After homogenization, the samples were cooled to room temperature over approximately 30 min. All measurements were conducted at 25 ± 0.2°C.

**Figure 1:**
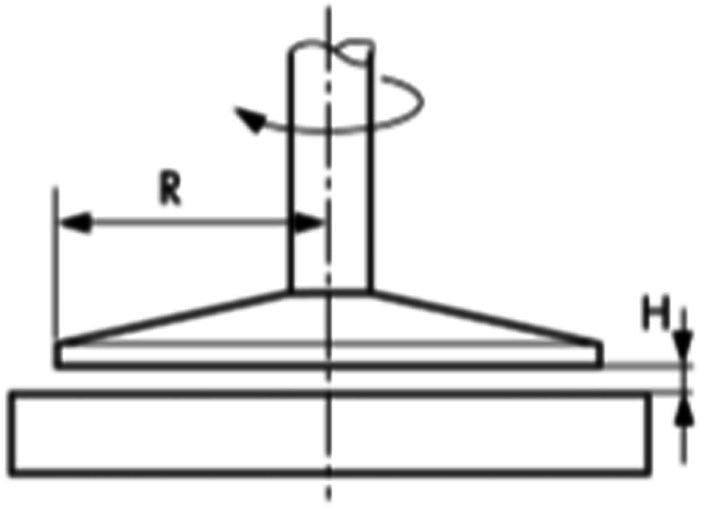
Measuring arrangement (24).

The rheological characteristic of the samples was measured with Anton Paar MCR302 type oscillation rheometer, both with oscillation and rotation rheometric methods. The measurements were done with PP50 measuring head, which is a 50 mm diameter stainless steel probe and we applied plane type geometry (Figure 1).

In this study, we employed two separate measuring techniques: amplitude sweep in oscillation mode and flow curb determination in rotation mode. According to the advice in the Mezger book (24) for industrial grease, we first applied some pre-test to determine the optimal measuring settings for the experiences. The following measurement techniques were used to analyze our samples based on these.

During the amplitude sweep measurements, we recorded the values of the storage modulus (G’) and loss modulus (G”) and identified the flow point and the linear viscoelastic region, if present, in the samples. The measurement setup included a gap height (H) of 1 mm, an angular velocity of 10 rad/s, and a shear deformation range of 0.001 to 1,000%.

To determine the flow curves of the fat mixtures, the samples were allowed to rest for 1 min after being placed in the sample holder. Prior to each analysis, the samples were pre-mixed at a steady-state shear rate of 100 s^−1^ for 1 min, followed by a 2-min rest period. The measurements began with an increasing shear rate ranging from 0.1 to 100 s^−1^, during which 60 data points were recorded. Subsequently, a steady-state shear rate of 100 s^−1^ was applied, with an additional 60 data points recorded. The average dynamic viscosity was calculated during the steady-state phase. Throughout the measurements, the shear-thinning or shear-thickening behavior of the samples was assessed. Model fitting, based on established literature, was employed to analyze the increasing shear rate portion of the data.

The most used models for the materials which has flow point are the Herschel-Bulkley (1.) and the Casson (2.) models (25–29). Therefore, these equations were used for model fitting.

1. Herschel-Bulkley model *τ* = *τ*_0_ + *η*_*pl*_ ∙ (*γ̇*)^*n*^
2. Casson model *τ*^0,5^ = *τ*_0_^0,5^ + *η*_*pl*_^0,5^ ∙ (*γ̇*)^0,5^

*τ*-shear stress [Pa], τ_0_-yield stress [Pa], η_pl_- plastic shear viscosity [Pas], *γ*-shear rate (1/s), t-time [s], n-flow index.

All measurements were repeated five times. The results were analyzed using Microsoft Excel, with model fittings performed through Excel Solver. To determine which sample exhibited the least deviation from the control, linear discriminant analysis (LDA) was employed.

## Results

### Amplitude sweep

**Figure 2:**
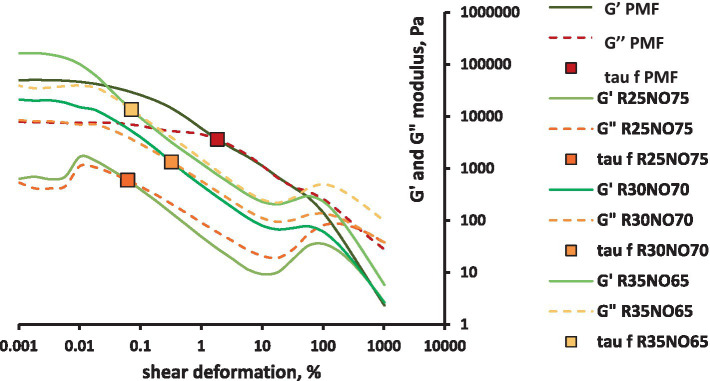
G’ and G” curves of fully hydrogenated rapeseed oil (R) and sunflower oil (NO) mixtures vs. shear deformation.

**Figure 3:**
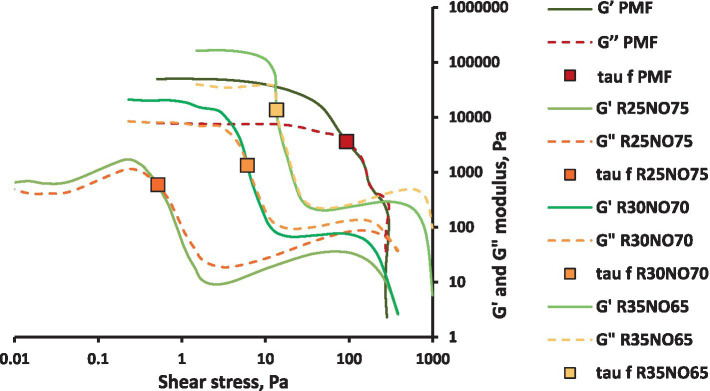
G’ and G” curves of fully hydrogenated rapeseed oil (R) and sunflower oil (NO) mixtures vs. shear stress.

The curves measured with amplitude sweep are shown in Figures 2, 3. The results showed that the PMF had a linear viscoelastic bound and a flow point. While investigating the rapeseed samples, we experienced that they have no linear viscoelastic bound, and the R25NO75 sample (composed of 25% fully hydrogenated rapeseed oil and 75% sunflower oil) showed very different behavior before the flow point. In case of the other two samples this uncertain interval did not occur. With increasing FH rapeseed ratio, the shear stress values in the flow point showed even bigger variability. The shapes of the curves are very different from the PMF curves. With increasing the FH rapeseed ratio, the G’ and the G” values are decreased and after the flow point a new maximum point can be seen in the curves. These waves are increasing with increasing the FH rapeseed amount in the sample. Further investigation is required to determine whether the observed wave patterns correlate with the fact that these samples are mixtures of components with different melting points or if other physical attributes play a role in wave formation. Focusing on the values at the flow point, it can be concluded that as the fully hydrogenated rapeseed oil content decreases, the shear stress values approach those of the control palm mid fraction (PMF). The results indicate that the sample with the highest fully hydrogenated rapeseed oil content (35%) is the most similar to PMF overall. However, at the flow point, the sample with the lowest fully hydrogenated rapeseed oil content (25%) shows the closest similarity in terms of texture and flowability.

### Rotational measurement

**Figure 4:**
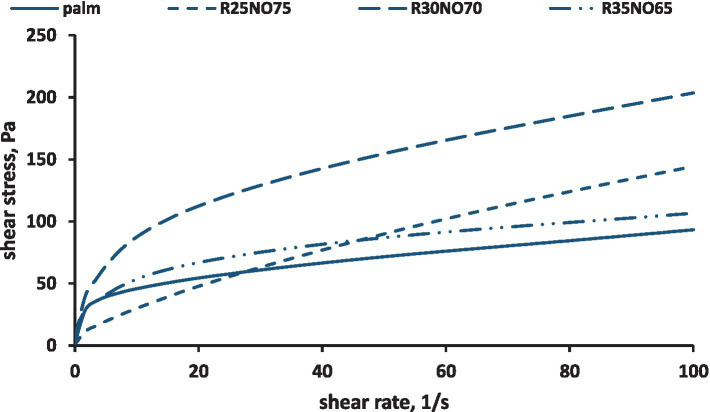
Measured shear stress values for different samples (R is fully hydrogenated rapeseed oil, NO is sunflower oil, “palm” is palm mid fraction).

During the rotational measurements, shear stress was recorded as shear rates were incrementally increased (Figure 4). As the shear rate increased, a corresponding rise in shear stress was observed, leading to the onset of flow in the samples. This behavior is characteristic of pseudoplastic flow, which may be attributed to the weakening of molecular interactions, allowing the molecules to realign relative to one another.

Our results indicated that the palm mid fraction (PMF) and the sample containing 35% fully hydrogenated (FH) rapeseed oil, and 65% sunflower oil exhibited the greatest similarity. The sample with 25% FH rapeseed oil and 75% sunflower oil had the steepest slope, meaning it began to flow more easily under increasing shear stress.

To accurately describe the rheological behavior, we applied rheological models, specifically fitting the Herschel-Bulkley and Casson models to the increasing shear rate portion of the flow curves. The parameters for the fitted models are presented in Table 1. The model that best fits the data is identified by a target cell value close to zero, an R^2^ value approaching one, and the smallest possible root mean square error (RMSE). Based on these criteria, the Herschel-Bulkley model provided a superior fit, particularly for the sample containing 25% FH rapeseed oil and 75% sunflower oil.

**Table 1:** The fitted models’ parameters.

		Herschel-Bulkley							Casson					
	Measured τ_0_	τ_0_	η_pl_	n	target cell	R^2^	RMSE	RMSE, %	τ_0_	η_pl_	target cell	R^2^	RMSE	RMSE, %
PMF 1	2.53	6.90	14.47	0.38	134.27	0.99	1.50	2.25	24.72	0.22	591.93	0.97	3.14	4.71
PMF 2	1.55	5.40	16.04	0.37	160.25	0.99	1.63	2.39	25.26	0.23	664.82	0.97	3.33	4.87
PMF 3	3.65	9.46	13.96	0.39	159.29	0.99	1.63	2.37	26.32	0.22	621.30	0.97	3.22	4.66
PMF 4	2.54	4.75	18.17	0.34	118.21	0.99	1.40	2.05	27.16	0.20	737.52	0.96	3.51	5.12
PMF 5	3.52	8.36	15.73	0.37	159.20	0.99	1.63	2.31	27.33	0.22	668.54	0.97	3.34	4.72
R25NO75_1	0.95	1.68	5.55	0.70	6.94	1.00	0.34	0.40	7.03	0.90	219.43	1.00	1.91	2.25
R25NO75_2	0.91	3.12	5.16	0.72	12.73	1.00	0.46	0.54	7.15	0.89	224.60	1.00	1.93	2.27
R25NO75_3	0.95	1.66	5.71	0.70	7.28	1.00	0.35	0.40	7.24	0.90	234.29	1.00	1.98	2.29
R25NO75_4	1.01	1.69	5.68	0.70	7.05	1.00	0.34	0.40	7.25	0.89	232.01	1.00	1.97	2.29
R25NO75_5	0.68	3.09	5.10	0.72	13.97	1.00	0.48	0.57	7.03	0.90	222.53	1.00	1.93	2.26
R30NO70_1	6.64	4.00	34.97	0.38	117.79	1.00	1.40	0.95	49.40	0.56	3720.39	0.97	7.87	5.34
R30NO70_2	6.39	3.52	34.80	0.38	114.06	1.00	1.38	0.94	48.71	0.57	3749.98	0.97	7.91	5.38
R30NO70_3	5.84	3.78	33.42	0.38	113.81	1.00	1.38	0.96	47.26	0.56	3566.35	0.97	7.71	5.35
R30NO70_4	5.33	2.24	34.96	0.38	118.54	1.00	1.41	0.96	47.83	0.57	3785.53	0.97	7.94	5.44
R30NO70_5	4.42	2.12	34.38	0.38	113.63	1.00	1.38	0.96	46.95	0.57	3715.49	0.97	7.87	5.48
R35NO65_1	7.84	5.01	16.76	0.33	74.01	1.00	1.11	1.77	25.93	0.17	857.38	0.95	3.78	6.01
R35NO65_2	9.18	6.47	20.92	0.32	59.26	1.00	0.99	1.30	32.23	0.20	1202.00	0.95	4.48	5.86
R35NO65_3	9.69	7.90	17.66	0.33	65.08	1.00	1.04	1.51	29.62	0.18	974.88	0.95	4.03	5.84
R35NO65_4	10.48	6.86	21.96	0.33	81.51	1.00	1.17	1.43	34.09	0.22	1363.44	0.95	4.77	5.84
R35NO65_5	8.78	6.99	19.70	0.35	54.55	1.00	0.95	1.21	31.60	0.23	1137.15	0.96	4.35	5.53

*Τ*-shear stess [pa], τ_0_-yield stress [pa], η_pl_- plastic shear viscosity [pas], *γ*-shear rate (1/s), t-time [s], n-flow index.

An additional criterion for selecting the appropriate model is the alignment between the predicted and measured *τ*₀ values. This comparison further confirms that the Herschel-Bulkley model offers a more accurate description of the flow curves. Within this model, the flow index “n” describes the flowability of a material; a value below 1 indicates pseudoplastic behavior, which was observed in our samples. A lower flow index corresponds to a flatter shear stress–shear rate curve, signifying that the material is less capable of flowing under pressure.

In our study, the sample containing 25% FH rapeseed oil had the highest flow index value. Samples with 30–35% FH rapeseed oil content most closely resembled the PMF. We conclude that as the FH rapeseed oil content increased, the flow index values decreased, correlating with the samples’ more solid texture.

**Figure 5:**
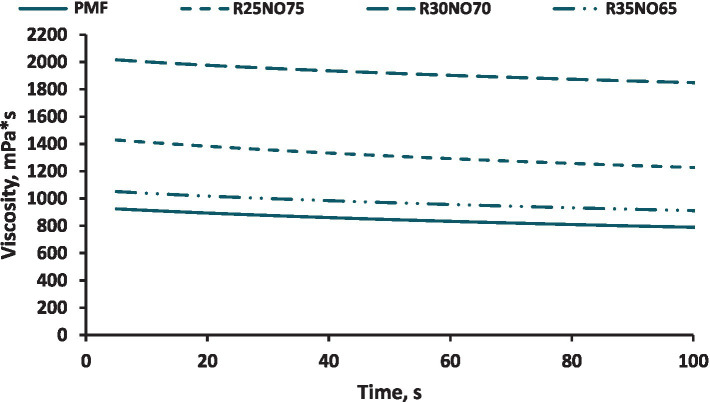
Viscosity curves of the different samples (R is fully hydrogenated rapeseed oil, NO is sunflower oil).

The dynamic viscosity values of the samples were also measured during the rotation measurement during the steady state shear rate interval. These allowed us to see that all of the samples’ viscosity values reduced over time (Figure 5), which indicates that all of the samples were shear thinning. According to our findings, the 35 percent FH rapeseed sample had viscosity values that were the closest to those of the PMF. It might be explained by the samples’ similar textures. The viscosity values were roughly 2.5 times higher for the sample containing 30% FH rapeseed fat and 1.5 times higher for the samples containing 25% FH rapeseed fat than the PMF.

### Linear discriminant analyses of the viscosity measurements

**Figure 6:**
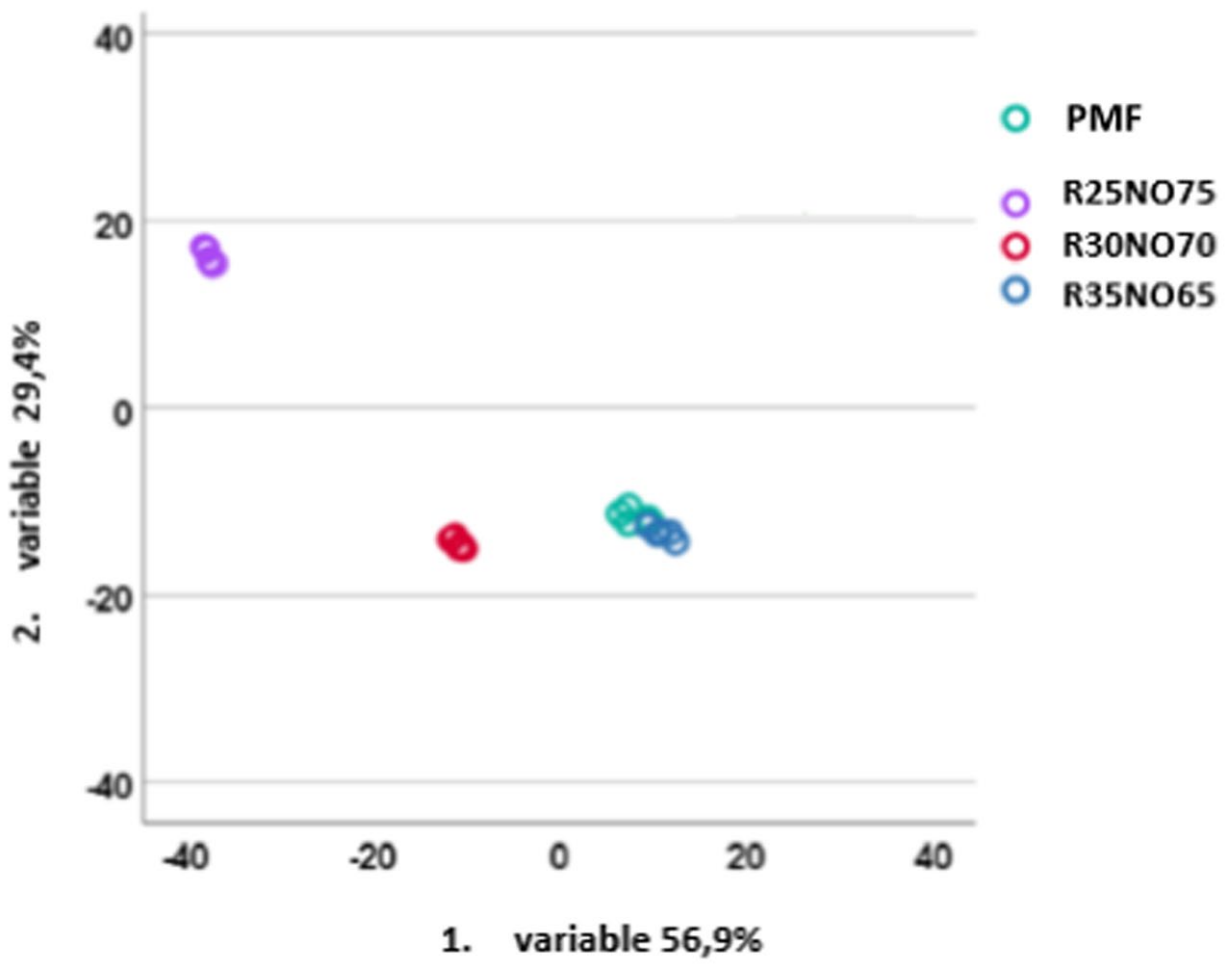
LDA results based on the rotational measurements parameters of the studied fat mixtures (R is fully hydrogenated rapeseed oil, NO is sunflower oil).

Linear discriminant analyses were used to distinguish the different samples from each other. The samples may be completely separated if all of the parameters from the two measurements are used. The same outcomes were obtained when we merely looked at the amplitude sweep parameters. However, the PMF samples and the 35 percent FH rapeseed samples were mixed if we solely used the Herschel-Bulkley model’s findings. The grouping was successful to a level of 96 percent, while the cross-validation was successful to a level of 88 percent, with the PMF and 35 percent FH rapeseed samples again being mixed. This was determined by looking at the combined effect of all the parameters that were determined for the rotational measurement. Of all variations, discriminant variable 1 explained 56.9%, while discriminant variable 2 explained 29.4% (Figure 6). We could observe that the distance from the PMF increased as the FH rapeseed content decreased.

## Conclusion

The results of the amplitude sweep test indicated that the control palm mid fraction sample exhibited a linear viscoelastic region, while the mixed samples (FH rapeseed and sunflower oil mixtures) did not demonstrate these characteristics. The curves of the mixed samples differed significantly from those of the control. As the proportion of the fully hydrogenated rapeseed oil increased, both the flow point and shear stress values rose. The shear stress–shear rate curves from the rotational measurements revealed that the sample containing 35% FH rapeseed oil, and 65% sunflower oil was most similar to the control palm mid fraction sample. To describe the rheological behavior, both the Casson and Herschel-Bulkley models were applied, with the Herschel-Bulkley model providing a better fit across all parameters. Given that the flow index was less than 1, this model confirmed that all samples exhibited pseudoplastic behavior.

Furthermore, the average dynamic viscosity values obtained from the steady-state shear rate portion of the measurements indicated that all samples displayed shear-thinning behavior. Among the samples, the 35% FH rapeseed oil sample was most similar to the control palm mid fraction in terms of viscosity readings.

Following the rheological measurement, we employed linear discriminant analysis to assess the distinctiveness of the sample groups. For accurate differentiation between groups, all criteria must be considered. However, when using only the Herschel-Bulkley model or the rotational measurement parameters, the 35% FH rapeseed oil sample and the palm fat sample were not entirely distinguishable. According to the conventional rotational viscometry method used in the industry, the difference between the control sample and the 35% FH rapeseed oil sample appears minimal. Nevertheless, other measurements might reveal more pronounced differences between the samples, suggesting that the choice of measurement technique could significantly impact the final outcomes. Therefore, a full circle assessment is needed when the task is to replace the used fat in the recipes.

Overall, the 35% FH rapeseed oil sample yielded results most closely resembling the control palm mid fraction. To fully assess the suitability of palm fat substitution, further research involving additional techniques such as texture analysis, thermoanalytical measurements, and organoleptic testing is essential.

## Data Availability

The raw data supporting the conclusions of this article will be made available by the authors, without undue reservation.
